# Unraveling the Role of Surface Mucus-Binding Protein and Pili in Muco-Adhesion of *Lactococcus lactis*

**DOI:** 10.1371/journal.pone.0079850

**Published:** 2013-11-18

**Authors:** Doan Thanh Lam Le, Thi-Ly Tran, Marie-Pierre Duviau, Mickael Meyrand, Yann Guérardel, Mickaël Castelain, Pascal Loubière, Marie-Pierre Chapot-Chartier, Etienne Dague, Muriel Mercier-Bonin

**Affiliations:** 1 Université de Toulouse; INSA,UPS, INP; LISBP, Toulouse, France; 2 INRA, UMR792 Ingénierie des Systèmes Biologiques et des Procédés, Toulouse, France; 3 CNRS, UMR5504, Toulouse, France; 4 CNRS, LAAS, Toulouse, France; 5 CNRS, ITAV-UMS3039, Toulouse, France; 6 Université de Toulouse, LAAS, Toulouse, France; 7 INRA, UMR1319 Micalis, Jouy-en-Josas, France; 8 AgroParisTech, UMR Micalis, Jouy-en-Josas, France; 9 Université de Lille1, Unité de Glycobiologie Structurale et Fonctionnelle, UGSF, Villeneuve d'Ascq, France; 10 CNRS, UMR 8576, Villeneuve d'Ascq, France; Institut Pasteur, France

## Abstract

Adhesion of bacteria to mucus may favor their persistence within the gut and their beneficial effects to the host. Interactions between pig gastric mucin (PGM) and a natural isolate of *Lactococcus lactis* (TIL448) were measured at the single-cell scale and under static conditions, using atomic force microscopy (AFM). In parallel, these interactions were monitored at the bacterial population level and under shear flow. AFM experiments with a *L. lactis* cell-probe and a PGM-coated surface revealed a high proportion of specific adhesive events (60%) and a low level of non-adhesive ones (2%). The strain muco-adhesive properties were confirmed by the weak detachment of bacteria from the PGM-coated surface under shear flow. In AFM, rupture events were detected at short (100−200 nm) and long distances (up to 600−800 nm). AFM measurements on pili and mucus-binding protein defective mutants demonstrated the comparable role played by these two surface proteinaceous components in adhesion to PGM under static conditions. Under shear flow, a more important contribution of the mucus-binding protein than the pili one was observed. Both methods differ by the way of probing the adhesion force, i.e. negative force contact vs. sedimentation and normal-to-substratum retraction vs. tangential detachment conditions, using AFM and flow chamber, respectively. AFM blocking assays with free PGM or O-glycan fractions purified from PGM demonstrated that neutral oligosaccharides played a major role in adhesion of *L. lactis* TIL448 to PGM. This study dissects *L. lactis* muco-adhesive phenotype, in relation with the nature of the bacterial surface determinants.

## Introduction

The digestive epithelium is covered with a protective mucus layer, regarded as a visco-elastic and permeable hydrogel. This layer serves as an ecological niche for commensal and probiotic bacteria, and plays a role in the defense against enteric bacterial infections by expelling pathogens from the mucosal surface [1]. The mucus layer is described as a secreted mucin-fiber scaffold [2]. Mucins are large glycoproteins with a serine and threonine-rich protein backbone, linked to a wide variety of O-linked oligosaccharide side chains arranged in a bottle-brush configuration [2]. Such O-glycans are nutritive sources for bacteria and/or potential ligands for bacterial adhesins [3], probably contributing in this way to the selection of the species-specific microbiota [4].

Many studies on bacterial muco-adhesion have been carried out with commensal *Lactobacillus* species, with the aim to select probiotics based on their ability to persist within the gut [5]–[8]. Several cell surface proteins have been shown to act as mediators of specific *Lactobacillus* adhesion to mucus [9]–[12]. By searching protein databases within different lactic acid bacteria (LAB) species, including lactococci and lactobacilli, Boekhorst *et al.* [13] and Kleerebezem *et al.* [14] concluded that the mucus-binding (MUB) repeat plays a pivotal role in host-bacteria interactions. Furthermore, the presence of pili structures and related muco-adhesion properties were reported in *Lactobacillus rhamnosus* GG [15].

*Lactococcus lactis*, considered as the model LAB, is traditionally used as a starter in manufacturing cheese and other fermented dairy products. Even though lactococci are not a frequent natural element of the intestinal microbiota, they were sporadically isolated from feces of many different groups of humans [16]. Certain strains were also shown to transit through the stomach and survive in the gut of rodents [17], [18]. To date, in contrast with lactobacilli, little is known about the structural and functional factors involved in lactococci muco-adhesion. The presence of only one single MUB-domain-containing protein was reported in the study depicted by Boekhorst *et al.* [13], in the genome of laboratory plasmid-free *L. lactis* strains. Nevertheless, Giaouris *et al.* [19] and Passerini *et al.* [20] highlighted the genetic, genomic and phenotypic diversity, such as surface physico-chemical properties, within large collections of *L. lactis* strains isolated from different ecological niches. Such biodiversity was further explored in terms of muco-adhesive ability. Indeed, we focused on elucidating interactions between *L. lactis* strains and a model mucin (pig gastric mucin (PGM)), using the natural strain *L. lactis* subsp. *cremoris* IBB477, isolated from a dairy environment and which was shown to persist *in vivo* in rat gut [17]. The higher adhesion force measured between IBB477 cells and PGM, compared to the control strain MG1820, was assessed at nanoscale with AFM force spectroscopy [21] and further confirmed at the bacterial population level, using quartz crystal microbalance with dissipation monitoring [22].

In the present contribution, we focused on unraveling multi-scale interactions between PGM and a vegetal *L. lactis* subsp. *lactis* isolate, TIL448, in close relation with the nature of the bacterial surface determinants involved. In a previous study, *L. lactis* TIL448 was shown to expose pili at its surface, conferring specific adhesion to Caco-2 human intestinal epithelial cells in contrast to the other tested *L. lactis* strains [23]. The gene cluster involved in pili synthesis is located on a plasmid and has the typical organization of genes specifying sortase-dependant heterotrimeric pili biosynthesis. Similarly to the gene cluster identified in the chromosome sequence of *L. lactis* IL1403 [24], the TIL448 plasmidic gene cluster encodes four putative proteins including a major pilin, a class-C sortase involved in major pilin polymerization, a minor pilin and a tip pilin. In addition, the presence of a mucus-binding protein, displaying two MucBP domains (PF06458) and which differs from the one identified by *in silico* analysis in the chromosome of sequenced *L. lactis* strains, was demonstrated at the surface of *L. lactis* TIL448. Noteworthy, this protein is also encoded by a plasmid-located gene.

In this framework, we performed single-cell scale AFM measurements with dedicated lacto-probes [25] and shear stress flow chamber experiments at the bacterial population level, under laminar flow conditions on the wild type *L. lactis* TIL448. We also tested the plasmid-cured strain (TIL1230) and two mutants (TIL1289 and TIL1290), obtained by disruption of the genes encoding the major pilin-encoding gene (*yhgE2*) and the mucus-binding protein (*muc*), respectively. Bacterial cells were put in adhesive contact with a biomimetic PGM-coated surface, under static or shear-flow conditions. Special attention was paid to the importance of specific interactions, as probed with AFM and the effect of variable flow conditions on shear-flow induced *L. lactis* detachment. Data of both sets of experiments were then combined to establish the role of surface mucus-binding protein and pili in *L. lactis* muco-adhesion, with a contribution closely depending on the static or dynamic nature of the environment and how interactions are probed.

## Materials and Methods

### Bacterial strains, plasmids, growth conditions and preparation of suspensions

The bacterial strains used in this study are listed in Table 1. Bacterial stock cultures were kept at −80°C in M17 broth (Oxoid), containing 0.5% (w/v) glucose and 20% (v/v) glycerol. Bacteria were first sub-cultured overnight at 37°C in M17-glucose (0.5% (w/v)) medium (M17Glc). This preculture was then used to inoculate M17Glc at 37°C. Erythromycin (5 µg/mL) was added when required. Bacteria were harvested during the exponential growth phase (optical density at a wavelength of 600 nm (OD_600_) of 1.2) by centrifugation (4000 rpm, 10 min, room temperature) and washed twice with MilliQ-grade water or phosphate buffered saline (PBS), according to further experiments to be performed (AFM and shear stress flow chamber, respectively).

**Table 1:** Bacterial strains used in this study.

Strain	Characteristics	Source
TIL448	*L. lactis* subsp. *lactis* NCDO2110, isolated from peas	INRA collection
TIL1230	Derivative of TIL448 obtained after curing plasmids by acridine orange treatment	M.-P. Chapot-Chartier^a^
TIL1289	Ery^R^; pilin mutant of TIL448 obtained by disruption of *yhgE2* gene encoding the major pilin, with the use of thermosensitive plasmid pGhost 9	M.-P. Chapot-Chartier^a^
TIL1290	Ery^R^; mucus-binding protein mutant of TIL448 obtained by disruption of *muc* gene encoding a mucus-binding protein, with the use of thermosensitive plasmid pGhost 9	M.-P. Chapot-Chartier^a^
TIL1295	Ery^R^; control strain TIL448 containing empty plasmid pGKV259	M.-P. Chapot-Chartier^a^

a Micalis, INRA, Jouy-en-Josas, France.

### Preparation of the PGM-coated polystyrene surfaces

The pig gastric mucin (PGM) was commercially available as a lyophilized powder (Sigma M1778, partially purified type-III mucin from porcine stomach). PGM was directly dissolved in PBS at pH 7.5 at a final concentration of 10 mg/mL. The solutions were prepared just before use.

Polystyrene was used as the substratum in the form of square (10.0 mm×10.0 mm×1.0 mm) or rectangular coupons (25.2 mm×6.3 mm×2.0 mm), for AFM and flow chamber experiments, respectively. Coupons were immersed in 2% (v/v) liquid detergent RBS 25 (Traitements Chimiques de Surfaces, Frelinghien, France) at 50°C for 15 min, rinsed in five successive baths of tap water at 50°C and five successive baths of tap water at room temperature, rinsed with copious amounts of MilliQ-grade water (50°C and room temperature) and finally air-dried for 15 min under a vertical flow hood (Cytosafe 2000, Faster, Italy). Surfaces were either used immediately for the following steps or stored in a desiccator at room temperature until use.

PGM adsorption onto polystyrene was performed as previously described [25] with slight modifications. Briefly, polystyrene coupons, prepared according to the above procedure, were exposed overnight to a 10 mg/mL PGM solution in PBS pH 7.5 at 4°C, under gentle agitation. After incubation, surfaces were copiously rinsed to remove loosely bound material using, in sequence, PBS and MilliQ-grade water and finally air-dried under a vertical flow hood (Cytosafe 2000, Faster, Italy).

The characteristics of the PGM layer after adsorption onto square polystyrene coupons have been described in details in a previous study [25]. Thus, before AFM force measurements, the shape of the water droplet partially wetting the PGM-coated surface was just compared to that obtained for the bare substratum in order to verify the enhanced wettability due to PGM adsorption. Reproducible results were obtained (data not shown). For rectangular polystyrene coupons, a more thorough surface analysis was carried out. First, surface wettability was evaluated before and after PGM coating, using the sessile drop technique with a Digidrop goniometer (Contact Angle Meter– GBX Scientific Instruments, Romans sur Isère, France), coupled with the WinDrop^++^ software to capture and analyse images. Reported values are the average of at least three advancing deionised water contact angle measurements per sample. Then, the physico-chemical properties of bare and PGM-coated surfaces were assessed with X-ray photoelectron spectroscopy (XPS), as previously detailed [25]. As for square coupons, before each experiment in the shear stress flow chamber, the shape of the water droplet formed on rectangular coupons was verified and a higher surface wettability due to PGM coating was systematically observed in a reproducible manner (data not shown).

### Isolation and separation of oligosaccharide-alditols from PGM

Commercial PGM was submitted to reductive β-elimination for 72 h at 37°C in 100 mM NaOH containing 1 M NaBH_4_. The reaction was stopped by the addition of cation exchange resin Dowex 50×8 (25–50 mesh, H^+^form) at 4°C until pH 6.5. After filtration on glass wool and evaporation to dryness, boric acid was eliminated by repetitive distillation as its methyl ester in the presence of methanol. The material was submitted to a cation exchange chromatography on Dowex 50×2 (200–400 mesh, H^+^ form) to remove residual peptides. The released O-glycans were desalted on Bio-Gel P2 column (Bio-Rad), eluted in water. Neutral and acidic O-glycans were separated by anion exchange chromatography fractionation on Dowex 1×2 (200–400 mesh, HCOO^−^ form). Neutral glycans were eluted from the resin by water and acidic glycans by stepwise concentrations of pyridine acetate (5 to 100 mM). Carbohydrate containing neutral and acidic fractions were pooled and desalted on Bio-Gel P2 column (Bio-Rad), eluted in water.

### Mass spectrometry analyses

MALDI-TOF mass spectra were acquired on a voyager Elite DE-STR mass spectrometer (Perspective Biosystems, Framingham, MA) in the reflection positive mode by delayed extraction using an acceleration mode of 20 kV, a pulse delay of 200 ns and grid voltage of 66%. Samples were prepared by mixing directly on the target 1 µL of oligosaccharide solution (1–5 pmol) with 1 µL of 2,5dihydroxybenzoic acid matrix solution (10 mg/mL in CH_3_OH/H_2_O, 50/50 v/v). Between 50 and 100 scans were averaged for each spectrum.

### AFM lacto-probe preparation

OTR4 (Si_3_N_4_) probes, purchased from Bruker Corporation (Palaiseau, France), were used for the lacto-probe preparation, as described elsewhere [25]. Briefly, cantilevers and tips, first cleaned for 15 min with UV/O_3_ treatment, were immersed for 5 hours in a polyethylenimine (PEI) solution (0.1% (w/v)), rinsed with a copious amount of MilliQ-grade water and stored under light vacuum. The negatively-charged *L. lactis* cells [19], suspended in MilliQ-grade water, were attached to the positively charged PEI-coated probes through a 20-min contact time. The presence of immobilized bacteria on the AFM tip was detected by scanning electron microscopy (SEM; Hitachi S-3700N). In addition, viability of attached cells was evaluated, as previously described [21]. In brief, the lacto-probe was first observed under bright field microscopy to visualize the total amount of immobilized cells. Then, the lacto-probe was labeled with carboxyfluorescein diacetate 100 µM (5(6)-CFDA: 5-(and-6)-carboxyfluorescein diacetate - mixed isomers, C195, Molecular Probes, 492−517 nm) for 1 h at 30°C, thoroughly rinsed with MilliQ-grade water, and reexamined under epifluorescence microscopy. Cells exhibiting an esterase activity are seen in green. Note that viability was checked on planktonic cells harvested at the exponential growth phase (complete CFDA labeling, data not shown). Control experiments were also performed with planktonic and attached dead cells after heat treatment (2 h, 95°C) and, as expected, no green fluorescence was detected (data not shown).

### AFM force spectroscopy

In order to ensure a firm immobilization of the *L. lactis* cells on the probe during the experiment, AFM measurements were performed in MilliQ-grade water and at room temperature, using the Catalyst system from Bruker Corporation (Santa Barbara, USA). Interactions between the lacto-probe and the PGM-coated polystyrene surface were assessed, for each strain tested, by recording at a loading rate of 161250 pN/s single force-distance curves and matrix of 32×32 force-distance curves on 5×5 µm^2^ squares, giving 1024 force curves to be analyzed. Experiments were performed with five independent PGM-coated surfaces and lacto-probes. For each lacto-probe, two zones (5×5 µm^2^) with homogeneous PGM coating [25] were probed. Blocking assays were performed with free PGM (10 mg/mL in PBS) and O-glycan fractions (total, acidic or neutral fraction, 10 mg/mL in PBS), prepared as described above. The spring constants of the tips, measured for each probe, were in the range 0.02 − 0.03 N/m. Adhesion forces were deduced from the force-distance curves and quantified using Research Nanoscope 8.31 software from Bruker Corporation. In brief, deflection data were recorded during the retraction of the tip from the PGM-coated surface and converted to force after multiplication with the spring constant of each individual cantilever, whereas the real tip-substrate distance was obtained by subtracting the deflection from the piezo movement. The point of zero distance was assigned at the intersection of the experimental curve with the straight line fitting the tail of the curve. Bond-rupture distances were deduced from the force vs. distance curves as the point of zero distance, subtracted from the point at which a negative deflection returns to zero.

### Detachment of *L. lactis* bacterial cells from PGM coating in the shear stress flow chamber

PGM-coated polystyrene coupons were subjected to shear flow-induced detachment experiments for each *L. lactis* strain under study. The experimental procedure, previously described for *Escherichia coli* in adhesive contact with plasma-modified stainless steel [26], was slightly modified, notably for the cell-counting mode. In brief, shear-flow induced *L. lactis* detachment was analyzed in a rectangular flow channel (12-mm width, 25.2-mm length and 200-µm thickness). The wall shear stress τ_W_ is given by: 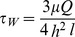



whereµ is the fluid dynamic viscosity (Pa.s), *Q* (m^3^/s) is the flow rate, *l* and *h* are respectively the channel half-width and half-thickness (m).

In order to have more "physiological" conditions for *L. lactis*, experiments were performed in PBS and at room temperature. The flow chamber and all tubes were filled with PBS, while care was taken to remove air bubbles from the system. The bacterial suspension (OD_600_ of 0.3, volume of 700 µL) was slowly injected into the flow chamber and bacterial cells were allowed to attach to PGM coating for 3 hours under “static” conditions. Images, collected using the reflection mode of an upright optical microscope (Nikon Eclipse LV100) equipped with a 40x ultra-long working distance objective, were recorded by a camera (digital STGHT DS-2MBW, Nikon) and the NIS-Elements F3.0 video acquisition software. The field of view was 144 µm by 108 µm with a resolution of 0.09 µm per pixel. Owing to previous results on velocity field in the flow chamber [27], special care was taken to properly choose the observation area, in order to satisfy requirements with respect to uniform flow conditions. Images were analyzed for estimating the percentage of the surface occupied by attached cells with the free software MacbiophotonicsImageJ (www.macbiophotonics.ca) and the Matlab software (Mathworks Inc., USA).

After the 3-h adhesion step, rinsing with PBS was achieved at a low flow rate of 0.001 mL/s (corresponding to a wall shear stress of about 0.012 Pa) in order to stabilize the system and remove loosely-adhering bacteria. The percentage of remaining attached cells was thereafter referred as to A_0_. We should note that the A_0_ value was in the range 1% − 3% of the total surface area, so that any interactions between neighboring bacteria were considered as minimal. Laminar flow of PBS was then imposed, with a stepwise increase in the flow rate (maximal value of 6.7 mL/s), with 3-min step duration. Flow rates ranging from 0.001 to 0.3 mL/s were generated by gravity, controlling through a toothed rack the height of a constant head vessel located upstream of the chamber. Higher flow rates were obtained using a gear pump (Ismatec, Fisher Bioblock Scientific). The maximal Reynolds number was equal to 560 (laminar flow conditions). The wall shear stress τ_W_ was in the range 0–80 Pa.

At the end of each step, the surface coverage by attached bacteria (A) was estimated. The detachment profile, representing the ratio A/A_0_ as a function of the wall shear stress τ_W_, was plotted. For each strain, experiments were performed at least in triplicate with different PGM-coated coupons and independently grown cultures.

## Results

### Using AFM for probing the adhesive properties of *L. lactis* TIL448 to PGM

**Figure 1:**
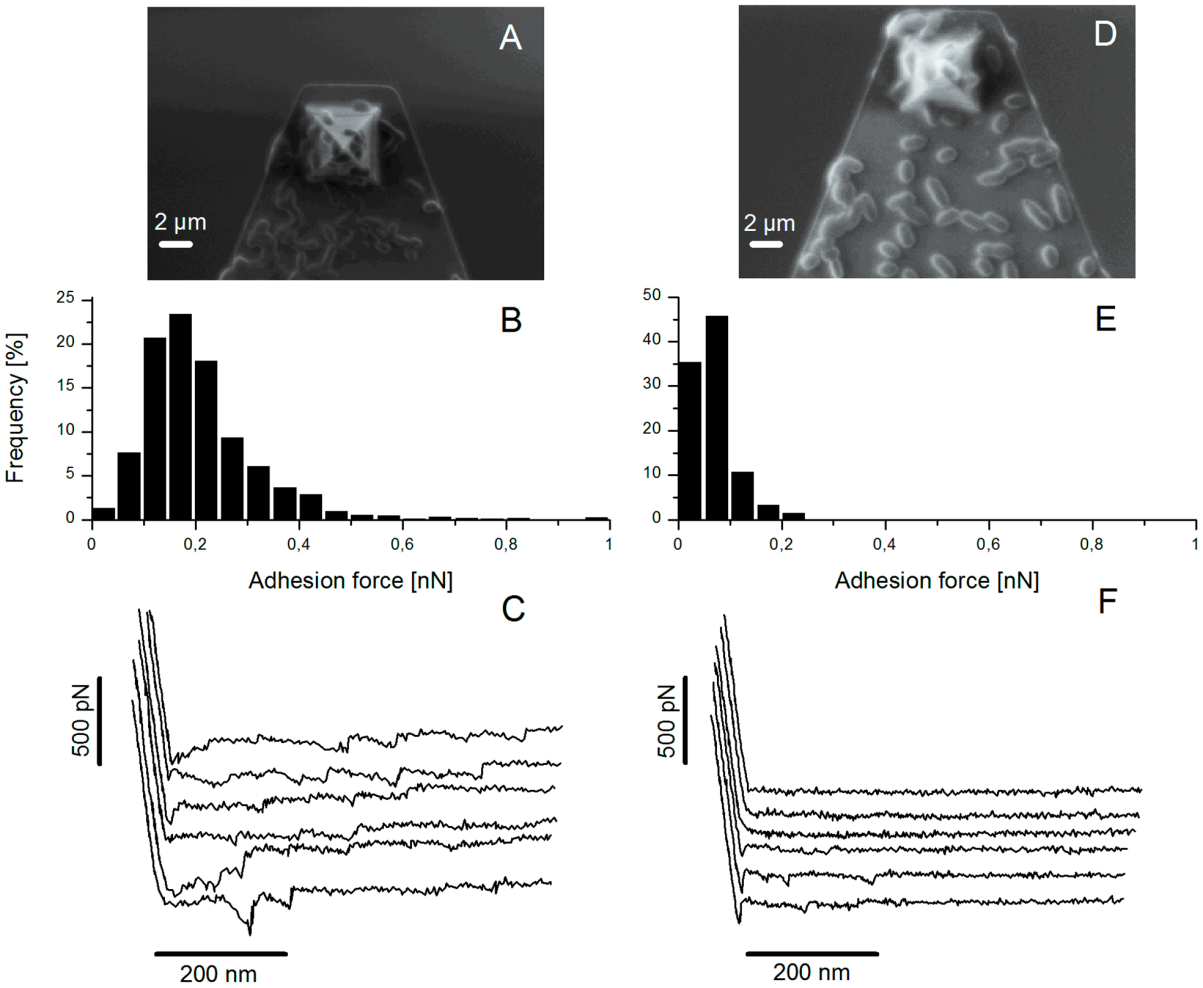
AFM force spectroscopy for the wild-type TIL448 and the plasmid-cured derivative TIL1230. (A, D) Representative SEM images of *L. lactis* bacterial cells immobilized onto AFM tip and cantilever; (B, E) histograms of adhesion forces and (C, F) typical force-distance curves obtained when probing interactions between the wild-type TIL448 (A, B, C) and the plasmid-cured derivative TIL1230 (D, E, F) and PGM-coated polystyrene using AFM force spectroscopy in milliQ-grade water. One representative experiment (1024 force curves) is shown.

A lacto-probe consisting of bacterial cells immobilized on the AFM tip was prepared with *L. lactis* TIL448 and examined by SEM (Figure 1A). We should note that small clusters of cells were typically attached to the AFM tip. To check cell viability, the lacto-probe was first observed under bright field microscopy (Figure S1A in File S1), then labeled with carboxyfluorescein diacetate (CFDA), for which cells exhibiting an esterase activity are seen in green, thoroughly rinsed with MilliQ-grade water and reexamined under epifluorescence microscopy. *L. lactis* bacterial cells on the probe were shown to be viable, or at least esterase active (Figure S1B in File S1). Furthermore, after 2-h AFM force measurements, cells were still viable (data not shown).

**Figure 2:**
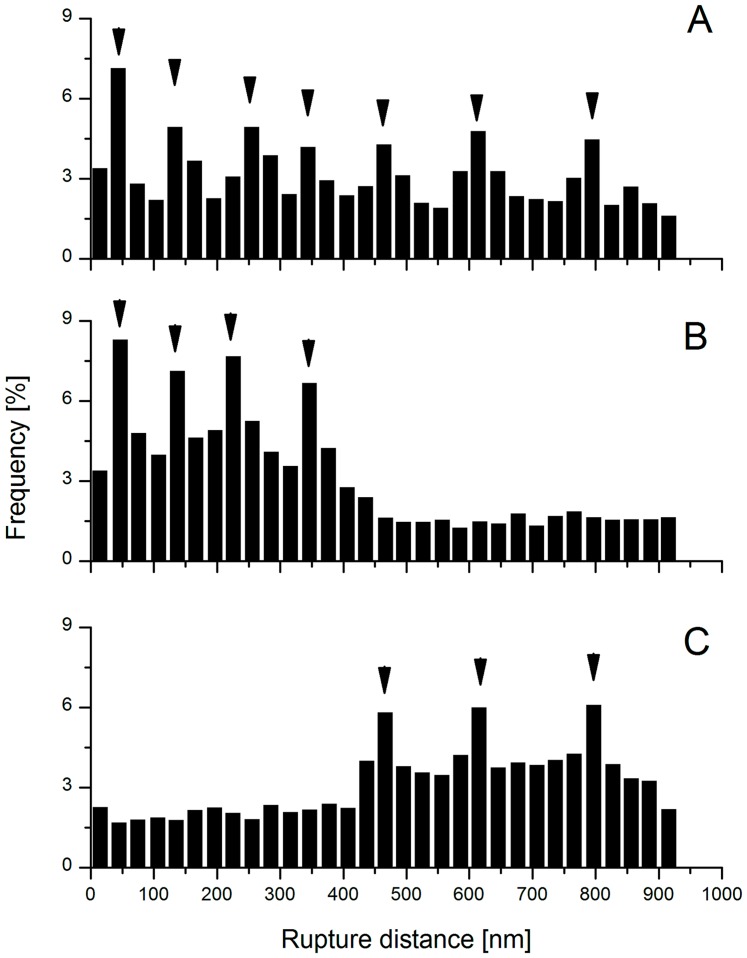
Rupture distances for the wild-type TIL448, the pilin mutant TIL1289 and the mucus-binding protein mutant TIL1290. Histograms of rupture distances corresponding to specific adhesive events for (A) wild-type TIL448; (B) pilin mutant TIL1289 and (C) mucus-binding protein mutant TIL1290 for one representative experiment (1024 force curves). Some typical rupture distances are indicated by arrows.

Histograms of adhesion forces together with typical force-distance curves obtained when probing interactions between TIL448 bacterial cells and PGM-coated polystyrene are displayed in Figures 1B and 1C, respectively. The adhesion force and the repartition of the percentages corresponding to non-adhesive, non-specific adhesive and specific adhesive events are also summarized in Table 2, for one representative experiment. The muco-adhesive properties of TIL448 were clearly assessed with a low level of non-adhesive events (only 2%) and a high proportion of specific ones (60%). An adhesion force of 0.18±0.04 nN was obtained. Rupture events were observed at short (100−200 nm) and long distances (up to 600−800 nm) (Figure 2A).

**Table 2:** Adhesion forces and percentage of non-adhesive, non-specific adhesive and specific adhesive events obtained using AFM force spectroscopy in milliQ-grade water on TIL448, TIL1230, TIL1289 and TIL1290 lacto-probes in contact with PGM-coated polystyrene.

		TIL448 (wild-type)	TIL1230 (plasmid-cured)	TIL1289 (pilin minus)	TIL1290 (mucus-binding protein minus)
**Force (nN)**		0.18±0.04	0.09±0.02	0.10±0.03	0.12±0.04
**Percentage of events**	NA^a^	2%	37%	31%	20%
	NSA^b^	38%	55%	43%	51%
	SA^c^	60%	8%	26%	29%

a NA: non-adhesive event.

b NSA: non-specific adhesive event.

c SA: specific adhesive event.

Noteworthy most *L. lactis* strains contain mobile genetic elements such as plasmids, responsible for the natural diversity among strains [28], [29]. Thus, genetic determinants for the observed muco-adhesive phenotype of *L. lactis* TIL448 (e.g. genes encoding surface proteins) could be located on plasmids that are present in the strain. To verify this hypothesis, a plasmid-cured derivative TIL1230 [23] was tested for its muco-adhesive properties by force spectroscopy. SEM image of TIL1230 lacto-probe, histograms of adhesion forces and typical force-distance curves are displayed in Figures 1D, 1E and 1F, respectively. Note that, as for TIL448, bacterial cells attached to the AFM probe were viable before and after 2-h force measurements (Figure S2 and Figure S3 in File S1, respectively). The adhesion force and the repartition of the percentages corresponding to non-adhesive, non-specific adhesive and specific adhesive events are indicated in Table 2. The plasmid-cured TIL1230 strain exhibited an adhesion force of 0.09±0.02 nN, 37% of non-adhesive events and only 8% of specific adhesive events, close to levels depicted for the low-adhesive control MG1820 [25]. Taken together, these results clearly demonstrated that the genetic determinants involved in the muco-adhesive phenotype of *L. lactis* TIL448 are plasmid-located.

### Assessing with AFM the role of surface proteins in adhesion of *L. lactis* TIL448 to PGM under static conditions

Two genes encoding surface proteins and located on plasmids were previously identified in TIL448 [23]. These genes, named *yhgE2* and *muc*, encode a pilin and a mucus-binding protein, respectively. These two genes were previously inactivated in TIL448, leading to mutant strains TIL1289 (pilin mutant, devoid of pili) and TIL1290 (mucus-binding protein mutant), respectively (Table 1).

First, as for the wild-type strain (TIL448) and the plasmid-cured derivative (TIL1230), AFM force spectroscopy measurements were carried out on TIL1289 and TIL1290 mutants. Since both of them are erythromycin-resistant (see Table 1), we checked that the presence of erythromycin in the culture medium had no effect on adhesion of TIL448 to PGM. To this end, AFM force spectroscopy measurements on PGM coating were performed with a control erythromycin-resistant derivative of TIL448 (TIL1295) (Table 1). The histogram of adhesion forces was similar to that of TIL448 grown in the absence of antibiotic (data not shown).

Adhesion force levels for TIL1289 (pilin-mutant) and TIL1290 (mucus-binding protein mutant) were lower than that of the parental strain TIL448 (Table 2). Furthermore, the percentage of specific adhesive events was substantially reduced (26% and 29% for TIL1289 and TIL1290, respectively vs. 60% for TIL448), which was correlated to a significant increase in the occurrence of non-adhesive events (31% and 20% for TIL1289 and TIL1290, respectively vs. 2% for TIL448) (Table 2). The equivalent percentage of specific adhesive events, recorded for both variants, was a clear indication that pili and mucus-binding protein equally contribute to the muco-adhesive properties of TIL448, as probed with “static” AFM.

To deeper investigate mechanisms involved, rupture distances extracted from specific adhesive events were scrutinized. Corresponding histograms are presented in Figure 2. Interestingly, for wild-type TIL448, rupture events were observed at short (100−200 nm) and long distances (up to 600−800 nm) (Figure 2A) whereas for the pilin mutant (TIL1289), long-distance adhesive events were suppressed (Figure 2B). In contrast, for the mucus-binding protein mutant (TIL1290), short-distance adhesive events disappeared (Figure 2C). According to these results, we hypothesize that short-distance events reflect interactions of the mucus-binding protein with PGM whereas long-distance ones are rather representative of pili involvement.

### Assessing with the shear-stress flow chamber the role of surface proteins in adhesion of *L. lactis* TIL448 to PGM under dynamic conditions

In parallel, to unravel the role of mucus-binding protein and pili under dynamic conditions, detachment of *L. lactis* from PGM coating was evaluated in a shear stress flow chamber, for the same strains as those tested above with “static” AFM force spectroscopy. Accordingly, bacterial cells attached to PGM coating were subjected to a stepwise increase in wall shear stress (0−80 Pa), under well-controlled hydrodynamics (laminar flow conditions). Cells are thus exposed to hydrodynamic drag and torque that both increase with applied wall shear stress [30]. The rate of bacteria removal directly correlates to their muco-adhesive behaviour.

The surface characteristics of the PGM-coated coupons were determined, in comparison with those of bare polystyrene. Surface wettability was first evaluated using the sessile drop technique. For the bare substratum, a water contact angle of 88.9°±2.9° was reached. After PGM adsorption, it dropped down to 73.7°±4.1°. An increase in hydrophilic properties was thus obtained, albeit at a lesser extent than that previously reported [25], probably due to differences in the elemental surface composition of polystyrene, as analyzed by X-ray photoelectron spectroscopy (data not shown). Then, XPS experiments were carried out to confirm the presence of adsorbed PGM. C1s and N1s core level spectra after PGM coating are displayed in Figure S4 in File S1. Two C1s contributions, other than those specific to polystyrene, were observed [25]: one peak at 286.4 eV associated to C-O and/or C-N bonds and another one at 288.4 eV assigned to COOH or CONH compounds. Both peaks were characteristic of chemical groups present in the protein core and the glycan side chains of PGM. The N1s signal, not detected for the bare substratum as expected. was composed of one peak at 400.4 eV, which was indicative of the PGM protein core [25]. Altogether, these results clearly demonstrated an efficient PGM adsorption onto polystyrene.

**Figure 3:**
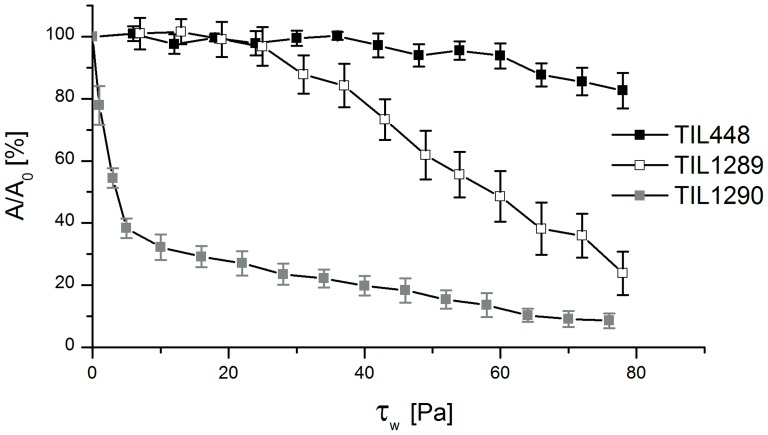
Detachment under shear flow of the wild-type TIL448, the pilin mutant TIL1289 and the mucus-binding protein mutant TIL1290. Shear-flow induced detachment profiles of *L. lactis* bacterial cells attached to PGM-coated polystyrene in PBS; wild-type TIL448 (black square); pilin mutant TIL1289 (white square) and mucus-binding protein mutant TIL1290 (light grey square). The given results are the average values and standard deviations over at least three different coupons and independently grown cultures.

Detachment profiles (i.e. normalized surface coverage vs. applied wall shear stress) of wild-type TIL448, plasmid-cured TIL1290 and mutant TIL1289 and TIL1290 strains from the PGM-coated surfaces are presented in Figure 3. We should note that the surface wettability was evaluated before and after a thorough PBS washing in the flow chamber in a stepwise manner, with a 3-min duration for each wall shear stress step, tested in the range 0−80 Pa. Water contact angle remained the same (data not shown), indicating that no PGM desorption occurred, despite the high wall shear stresses applied. Detachment data were interpreted by evaluating three representative parameters [26]: τ_W50%_ (wall shear stress needed to remove 50% of the bacterial cells initially attached to PGM coating), τ_W5%_ (threshold value for bacterial cell detachment) and τ_W90%_ (for evaluating the ability of quasi-complete detachment). Values obtained for all strains under study are reported in Table 3.

**Table 3:** τ_W5%_, τ_W50%_ and τ_W90%_ values (Pa) obtained in the shear stress flow chamber for *L. lactis* bacterial cells attached to PGM coating in PBS.

	TIL448 (wild-type)	TIL1289 (pilin minus)	TIL1290 (mucus-binding protein minus)	TIL1230 (plasmid-cured)
**τ_W5%_ (Pa)**	10.1±0.4	12.9±12.0	0.2±0.1	No adhesion
**τ_W50%_ (Pa)**	Not reached	56.8±8.6	4.0±2.2	No adhesion
**τ_W90%_ (Pa)**	Not reached	Not reached	62.2±2.4	No adhesion

The given results are the average values and standard deviations over at least three different PGM-coated coupons and independently grown cultures.

Shear-flow induced detachment of *L. lactis* from PGM coating was shown to be strongly strain-dependent (Figure 3). Wild-type TIL448 strain exhibited high PGM-adhesive properties, in contrast with its plasmid-cured derivative TIL1230, which was not able to adhere to PGM-coated polystyrene, even after 3-h contact time (data not shown). Indeed, for TIL448, the fraction of detached bacteria was low, even for increasing wall shear stress and, at the end of the experiment with a maximal wall shear stress of about 80 Pa, nearly 80% of the initial bacterial population remained attached to PGM coating (Figure 3). Such results are in good agreement with those previously described at the single-cell level and static conditions with AFM (see above). We also confirmed that the control TIL1295 exhibited the same muco-adhesive properties as TIL448 (data not shown).

Moreover, the respective role of *L. lactis* surface determinants involved in muco-adhesion was elucidated with the shear stress flow chamber, considering TIL1289 and TIL1290 mutants. Both mutants exhibited weaker adhesion than that of the wild type (Figure 3). Nevertheless, detachment profiles were quite different. Indeed, the fraction of detached cells was high for TIL1290 (mucus-binding protein mutant) throughout the experimental range whereas, for TIL1289 (pilin-mutant), cell removal was significantly lower. This was confirmed by the analysis of τ_W5%_, τ_W50%_ and τ_W90%_ values (Table 3). Indeed, τ_W5%_ was the lowest for TIL1290 (i.e. no threshold) with close values for TIL448 and TIL1289 (0.2±0.1 Pa against 10.1±0.4 Pa and 12.9±12.0 Pa for TIL1290, TIL448 and TIL1289, respectively) whereas, as expected, τ_W50%_ was intermediate for TIL1289 (56.8±8.6 Pa vs. 4.0±2.2 Pa for TIL1290, not reached for TIL448). τ_W90%_ reached 62.2±2.4 Pa for TIL1290 (not reached for TIL1289 and TIL448). Taken together, these results indicate a more important contribution of the mucus-binding protein than pili in the muco-adhesive phenotype of *L. lactis*, as probed under shear flow.

### Deciphering with AFM the role of mucin O-glycans in adhesion of *L. lactis* TIL448 to PGM

**Figure 4:**
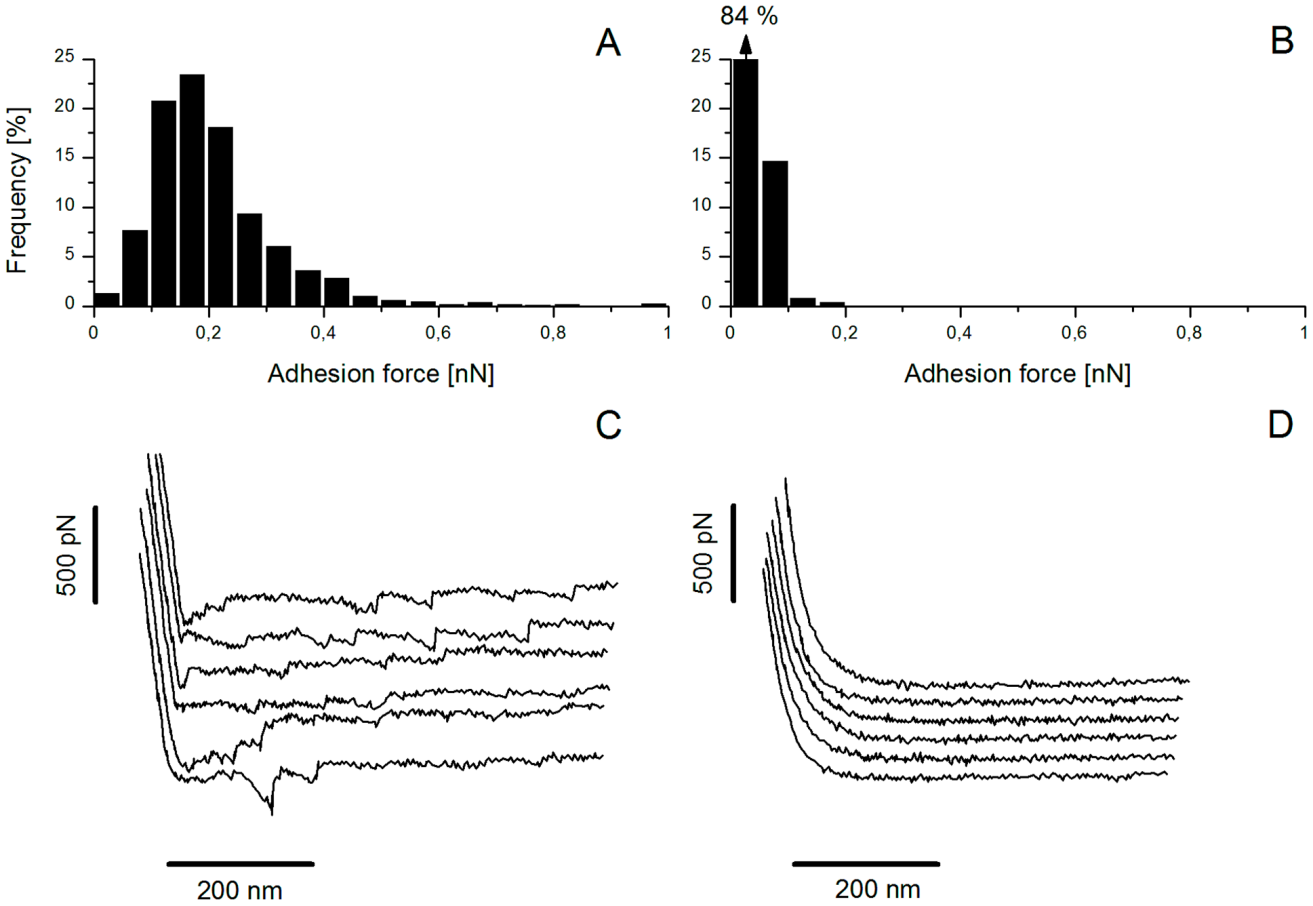
AFM blocking assays with free PGM for the wild-type TIL448. (A, B) Histograms of adhesion forces and (C, D) typical force-distance curves for wild-type TIL448 lacto-probe in interaction with PGM-coated polystyrene, using AFM force spectroscopy in milliQ-grade water, before (A, C) and after (B, D) incubation with free PGM. One representative experiment (1024 force curves) is shown.

In order to get a deeper insight into the specificity of the adhesive events depicted for *L. lactis* TIL448, AFM blocking assays were carried out, first with free PGM (Figure 4). Subsequently, the TIL448 lacto-probe was incubated in PGM solution, before putting it in contact with the PGM-based coating. The number of curves showing adhesive events as well as the measured binding forces were dramatically reduced, indicating that the adhesion forces measured using the lacto-probe were specific to the *L. lactis*/PGM interaction. Indeed, on one representative experiment, the percentage of non-adhesive events substantially increased, from 2% to 84% (Figures 4A and 4B). The shape of force-distance curves after the lacto-probe incubation with free PGM (Figure 4D) confirmed the presence of non-adhesive events.

On this basis, further blocking assays were performed with total, acidic and neutral fractions of O-glycans, after their isolation and purification from PGM. Percentages of non-adhesive events after blocking with each tested fraction are displayed in Table 4. A substantial increase, compared to before-blocking conditions (2%, see above), was systematically observed. Indeed, inhibition of adhesion with total O-glycans (76±5%) was of the same order of magnitude as the value reported for free PGM (80±4%). Moreover, the neutral oligosaccharidic fraction exerted a higher blocking effect than the acidic one (42±10% vs. 11±5%) (Table 4).

**Table 4:** AFM blocking assays with PGM or O-glycan fractions (total, acidic, neutral) purified from PGM for *L. lactis* TIL448 lacto-probe in contact with PGM-coated polystyrene in milliQ-grade water.

Solution	PGM	Total O-glycans	Acidic fraction	Neutral fraction
% NA^a^ after blocking	80±4%	76±5%	11±5%	42±10%

The given results are the average values and standard deviations over five independent PGM-coated coupons and lacto-probes. Before blocking, the percentage of non-adhesive events for *L. lactis* TIL448 was 2% (see in the Results section).

a NA: non-adhesive events.

## Discussion

The lacto-probe concept and the associated AFM measurements were applied to *L. lactis* subsp. *lactis* TIL448 and its derivatives for probing interaction mechanisms with the model mucin PGM. The shear stress flow chamber was then implemented for monitoring the shear-flow induced detachment of the same panel of *L. lactis* strains under laminar flow conditions. Data derived from a single-cell scale “static” method (AFM) vs. a multi-cellular scale “dynamic” one (flow chamber) were combined to establish the role of mucus-binding protein and pili in *L. lactis* muco-adhesion.

For TIL448, an adhesion force of 0.18±0.04 nN was reached, which is consistent with the value previously reported for *L. lactis* subsp. *cremoris* IBB477 (0.22±0.05 nN) [21]. Further, such values may be related to the adhesive force recently reported for the specific interactions between a lectin-coated surface and the cell surface glycopolymers of the probiotic *Lactobacillus plantarum* (0.251±0.146 nN) using single-cell force spectroscopy [31]. Moreover, as previously reported for *L. lactis* subsp. *cremoris* IBB477 [21], adhesion of *L. lactis* TIL448 to PGM was driven by an interplay between specific and non-specific forces. During AFM force spectroscopy, three representative curve shapes were obtained, as shown in Figure S5 in File S1: (1) no adhesive event detected upon retraction of the tip from the PGM-coated surface; (2) non-specific adhesive events (showing no extension before rupture) corresponding to hydrophobic, electrostatic, or Lifshitz-van der Waals interactions; and (3) one, two, or three specific adhesive events, occurring at several nanometers after the contact point. Such multiple adhesive events, attributed to specific interactions involving ligand-receptor bonding, like here cell surface protein/sugar complexes [21], were recently more deeply investigated using AFM tips functionalized with different types of lectin, in contact with purified PGM [32]. For TIL448, 60% of the force curves were assigned to specific adhesive events, which was significantly higher than the value previously reported for IBB477 (20% [21]). We should also note that the inhibition percentage achieved during blocking tests with total O-glycans was higher than that obtained for IBB477 (mean value of 76% against 48%, for TIL448 and IBB477 [21], respectively), probably due to differences between these two strains in terms of type and characteristics of cell surface proteins involved in muco-adhesion. However, no data are to date available for *L. lactis* IBB477.

With the use of two targeted defective-mutants, the role of both mucus-binding protein and pili in adhesion of TIL448 to PGM was clearly established. Their contribution was shown to be equivalent, as “sensed” under AFM static conditions, since nearly identical percentages of specific adhesive events were reached (26% and 29% for the pilin and mucus-binding protein mutants, respectively). This conclusion is reinforced by the adhesion force values which were quite similar between both mutants (0.10±0.03 nN and 0.12±0.04 nN for the pilin and mucus-binding protein mutants, respectively), in comparison with the wild-type strain (0.18±0.04 nN). Such force levels are consistent with the mean rupture force of 95 pN, reported for type-IV piliated *Pseudomonas aeruginosa* cell probe in contact with mica [33]. Elsewhere, the retraction forces for type-IV pili of *Neisseria gonorrhoeae*, as determined by optical tweezers, reached 0.11±0.03 nN [34].

All these results were supported by the further analysis of the bond-rupture distance, which is defined as the distance from the start of retraction to the point of final rupture. This essentially corresponds to the distance between the AFM tip and the substrate when the ligand-receptor complex is fully extended. For wild-type TIL448, we observed rupture events at short (100–200 nm) and long distances (up to 600–800 nm). The short rupture distances, observed with the wild-type strain and further confirmed with the pilin mutant (TIL1289), undoubtedly reflect interactions between the mucus-binding protein present on the *L. lactis* cell wall with sugars of PGM (see below). The existence of long rupture distances, also detected with the mucus-binding protein mutant (TIL1290), is indeed consistent with the elongated architecture of pili [35], [36]. In the AFM study probing interactions between type-IV piliated *P. aeruginosa* and mica surface [33], the authors observed long rupture distances (up to 1 µm). When they tested the non-piliated mutant, rupture lengths were drastically reduced (10 to 100 nm), which is fully consistent with our own results. The presence of pili and related muco-adhesion properties were previously reported in *L. rhamnosus* GG [15], [37]. Such properties are expected to confer to *L. rhamnosus* GG a distinct advantage over non-piliated probiotics for maintaining stable, or at least, extended residence within the gastrointestinal tract [37].

Parallel to AFM measurements, muco-adhesion of *L. lactis* was evaluated under well-defined flow field conditions, which are more relevant from a physiological point of view. Dynamic conditions are indeed encountered in many compartments within the gastrointestinal tract (e.g. shear fluctuations caused by salivary washing or intestinal peristalsis [38]). The effect of shear stress on the adhesion of bacteria, yeast cells and colloidal particles has been addressed in numerous works, using specially-dedicated laminar flow chambers. A decrease in the total number of adhesion events as a function of shear has often been depicted [26], [30], . However, to our knowledge, data on the use of flow-induced shear forces to probe bacterial muco-adhesion are scarce. Likewise, no study has previously evaluated how muco-adhesion may be modulated by cell surface proteins (as here, mucus-binding protein and pili), separately in mutants and in combination within the wild-type strain. Indeed, we observed that an increase in the wall shear stress facilitated *L. lactis* bacterial cell detachment, albeit at a variable extent depending on the strain under study. In contrast with AFM results, a more important contribution of the mucus-binding protein than pili in the muco-adhesive phenotype of *L. lactis* was assessed under shear flow. Nevertheless, worth mentioning is the different mode used for both methods for probing the force, as pointed out by Xu *et al*. [42]: with AFM, the tip of the cantilever pushes the bacteria cells against the surface and then bring them away, normal to the surface. In contrast, the flow-chamber method operates under “mild conditions” using the drag force to detach cells, parallel to the surface, mainly *via* rolling [27]. During AFM measurements, all the individual surface determinants acting against detachment are stressed together while, with the second method, only a part of them located at the trailing edge of the bacterial cell dissociate, described as a peeling process [43]. The weaker adhesion under shear flow of the piliated mutant (TIL1290), compared to its non-piliated counterpart (TIL1289), could be related to the presence of long pili (rupture events up to 600−800 nm, as found with AFM), which prevented, through steric repulsions, maximum contact of *L. lactis* bacterial cells with PGM coating. In their study on adhesion of the Gram-negative *Xylella fastidiosa* bacteria (possessing both type-I and type-IV pili) to glass, based on the use of a microfluidic flow chamber in conjunction with the wild-type strain and two pilus-defective mutants, De la Fuente *et al.* [44] reported the tendency of the mutant with the longer type-IV pili to require lower drag forces for detachment from the substratum compared to results obtained with the other mutant only exhibiting short type-I pili. However, in the case of Gram-positive bacteria, pili have been shown to be extremely flexible [45] albeit inextensible structures [46]. Therefore, they would not be a large obstacle to get close to the substratum but could not withstand the drag force due to their inextensibility. Another possible explanation of weak adhesion of the piliated mutant under shear flow would be the low density of pili present at the cell surface for TIL448 [23]. The wild-type TIL448 adhered much better to PGM than the two mutants, showing that the co-expression of both short and long structures is required, as was alluded to previous work [44].

In addition, blocking assays with free PGM or with total, acidic and neutral fractions of O-glycans purified from PGM demonstrated that neutral oligosaccharides play a major role in interactions between PGM and *L. lactis* TIL448, with a percentage of non-adhesive events of 42±10% after blocking against 11±5% for the acidic fraction. We established the profiles of neutral and acidic O-glycans isolated from PGM. As revealed by MALDI-MS analysis of the released glycans, PGM is substituted by a complex mixture of neutral and sulfated glycans, with size ranging from two to twelve monosaccharides (Tables 5 and 6). Nordman *et al.* [47] showed that pig gastric mucus contains a number of distinctly different mucin populations varying in buoyant density, size, “acidity”, glycosylation, sulphation and tissue origin. Furthermore, the glycan composition that we observed is fully in agreement with previous studies reporting the prevalence of LacNAc-based O-glycans partially fucosylated in α1,2 on Gal residues and sulfated in 1,6 position of GlcNAc residues [48], [49].

**Table 5:** Composition of the neutral fraction of O-glycans released from PGM. Monosaccharide composition of individual glycans was established by MALDI-TOF-MS based on the *m/z* values of monosaccharides.

m/z	GalNAc-ol	Gal	GlcNAc	Fuc
408	1	1	0	0
449	1	0	1	0
554	1	1	0	1
611	1	1	1	0
757	1	1	1	1
773	1	2	1	0
814	1	1	2	0
919	1	2	1	1
976	1	2	2	0
1017	1	1	3	0
1065	1	2	1	2
1122	1	2	2	1
1138	1	3	2	0
1179	1	2	3	0
1284	1	3	2	1
1325	1	2	3	1
1430	1	3	2	2
1471	1	2	3	2
1487	1	3	3	1
1544	1	3	4	0
1585	1	3	5	0
1633	1	3	3	2
1649	1	4	3	1
1690	1	3	4	1
1706	1	4	4	0
1747	1	3	5	0
1795	1	4	3	2
1836	1	3	4	2
1852	1	4	4	1
1894	1	3	5	1
1910	1	4	5	0
1942	1	4	3	3
1999	1	4	4	2
2040	1	3	5	2
2056	1	4	5	1
2114	1	2	4	5
2161	1	5	4	2
2202	1	4	5	2
2217	1	5	5	1
2260	1	4	6	1

Nature of monosaccharides (Gal, GlcNAc and Fuc) was based on GC composition analysis and on previous reports [48]. *m/z* values of neutral glycans correspond to [M+Na]^+^ adducts.

**Table 6:** Composition of the acidic fraction of O-glycans released from PGM.

m/z	GalNAc-ol	Gal	GlcNAc	Fuc	Neu5Ac	SO_3_
902	1	1	1	0	1	0
1078	1	2	2	0	0	1
1021	1	2	1	1	0	1
1224	1	2	2	1	0	1
1281	1	2	3	0	0	1
1370	1	2	2	2	0	1
1386	1	3	2	1	0	1
1427	1	2	3	1	0	1
1532	1	3	2	2	0	1
1573	1	2	3	2	0	1
1589	1	3	3	1	0	1
1735	1	3	3	2	0	1
1751	1	4	3	1	0	1
1898	1	4	3	2	0	1
1955	1	4	4	1	0	1

Monosaccharide composition of individual glycans was established by MALDI-TOF-MS based on the *m/z* values of monosaccharides.

Nature of monosaccharides (Gal, GlcNAc and Fuc) was based on GC composition analysis and on previous reports [48]. *m/z* values of sialylated glycans correspond to [M+Na]^+^ adducts whereas sulphated glycans exhibited [M+2Na-H]^+^ adducts.

In agreement with the above findings, the TIL448 plasmid-encoded tip pilin, probably located at the extremity of the pili appendages, contains a lectin-type domain (PF00139 or *Lectin_leg* domain) characteristic of leguminous lectins, which bind typically hexoses and/or hexosamines [50], as found here for the neutral O-glycan fraction of PGM (Table 5). In addition, since fucose was identified in many of the neutral oligosaccharides (Table 5), it could be involved in the interactions between PGM and the mucus-binding protein of *L. lactis* TIL448. To support this hypothesis, in a previous work on *Lactobacillus reuteri* [9], the mucus-binding protein was shown to recognize carbohydrate structures in mucus and fucose could be part of the recognized structure. Further, the Fuc-binding lectin (*Ulex europaeus* agglutinin I (UEA)) was found to exhibit the strongest affinity for purified PGM compared to Gal-binding lectins (namely, *Ricinus communis* agglutinin I (RCA) and peanut (*Arachis hypogaea*) agglutinin (PNA)) [32].

In conclusion, this work was focused on the muco-adhesive phenotype of the vegetal isolate *L. lactis* subsp. *lactis* TIL448 at multi-scale, by coupling AFM force spectroscopy and shear stress flow chamber. Using the wild-type strain, in conjunction with pilin and mucus-binding protein defective mutants, the combined role played by both surface proteins was established, with a contribution closely depending on how interactions are probed. The importance of the sugar receptors of PGM, mainly of neutral type, was also demonstrated. The need for well-controlled hydrodynamics was highlighted, especially when shear-flow sensitive appendages like pili are involved. We hope that these findings on *L. lactis* muco-adhesion will lead to a better understanding of interactions of this LAB with the mucosal environment in the gastrointestinal tract, further offering novel strategies for medical and food-related applications.

## Supporting Information

File S1 **Supplementary Data**
(DOCX)
