# Supplementary material for: Unraveling the Role of Surface Mucus-Binding Protein and Pili in Muco-Adhesion of Lactococcus lactis
Source: PLoS One. 2013 Nov 18;8(11):e79850. doi: 10.1371/journal.pone.0079850 (PMC3832589; doi:10.1371/journal.pone.0079850)
Supplement: File S1 — Supplementary Data (DOCX) [file pone.0079850.s001.docx]

**FILE S1. Supplementary data**

**Figure S1. Fluorescence image of the TIL448 lacto-probe**

Fluorescence images of the biologically functionalized AFM probe, for *L. lactis* TIL448 cells immobilized onto AFM tip and cantilever. (A) Bright-field microscopy image of the TIL448 lacto-probe (total bacterial cells attached). (B) Fluorescence image of the TIL448 lacto-probe after CFDA labeling. Viable cells are seen in green. Scale bar: 20 µm.

**Figure S2. Fluorescence image of the TIL1230 lacto-probe**

Fluorescence images of the biologically functionalized AFM probe, for *L. lactis* TIL1230 cells immobilized onto AFM tip and cantilever. (A) Bright-field microscopy image of the TIL1230 lacto-probe (total bacterial cells attached). (B) Fluorescence image of the TIL1230 lacto-probe after CFDA labeling. Viable cells are seen in green. Scale bar: 20 µm.

**Figure S3. Fluorescence image of the TIL1230 lacto-probe after 2-h force measurements**

Fluorescence images of the biologically functionalized AFM probe, for *L. lactis* TIL1230 cells immobilized onto AFM tip and cantilever, and imaged after 2 hours of force measurements. (A) Bright-field microscopy image of the TIL1230 lacto-probe (total bacterial cells attached). (B) Fluorescence image of the TIL1230 lacto-probe after 2-h force measurements and CFDA labeling. Scale bar: 20 µm.

**Figure S4.** **XPS spectra for PGM-coated polystyrene surface**

(A) C1s core-level spectrum and (B) N1s core-level spectrum recorded by XPS for PGM-coated polystyrene surface. Chemical groups representative of PGM (protein core and glycan side chains) are indicated, together with their binding energy.


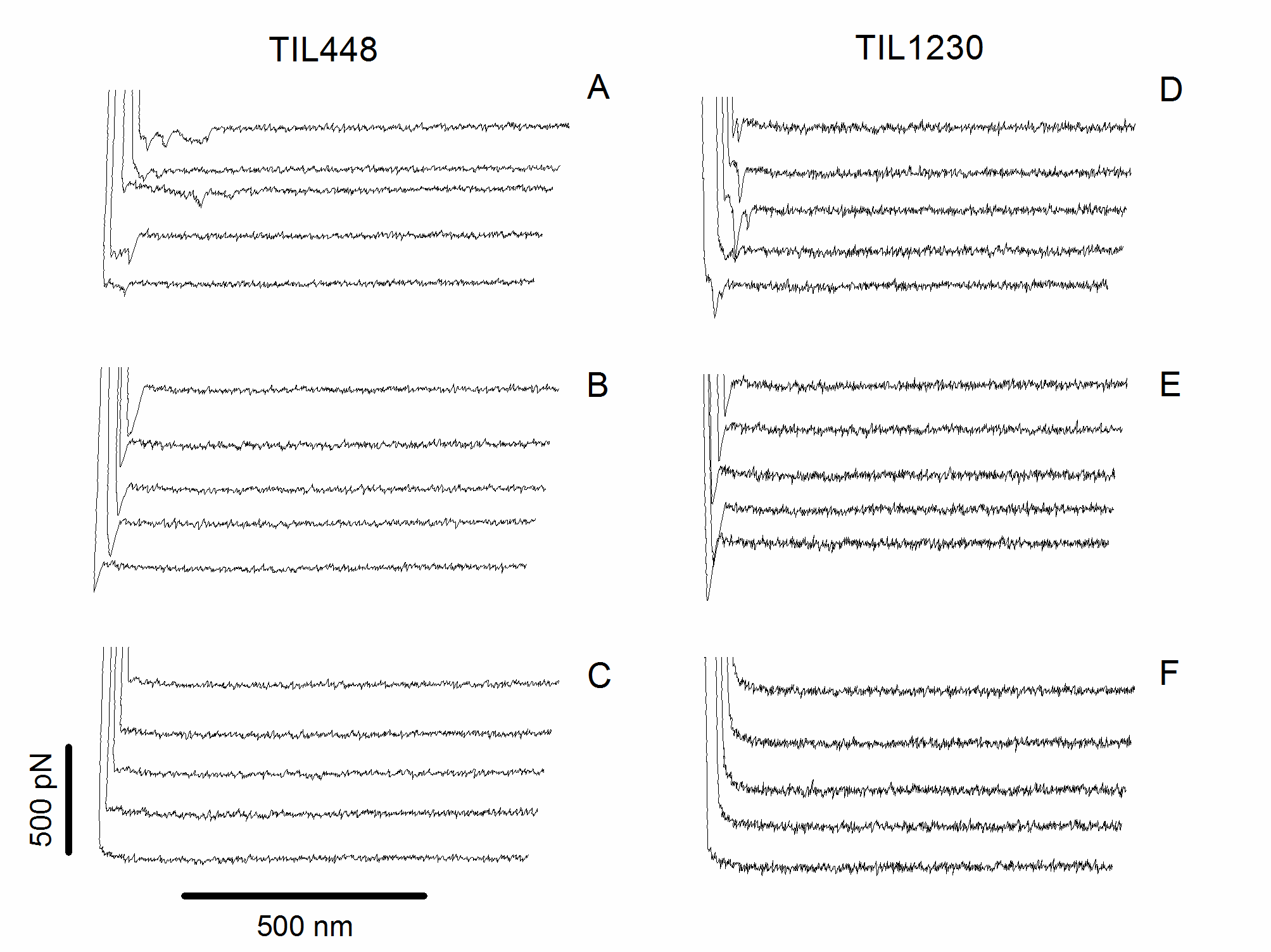


**Figure S5. Force curves for *L. lactis* TIL448 and TIL1230 lacto-probes after contact with PGM coating**

Five representative force curves for *L. lactis* TIL448 (A-C) and TIL1230 (D-F) lacto-probes after contact with PGM-coated surfaces for each condition (specific adhesion, non-specific adhesion and no adhesion): (A and D) specific adhesive events; (B and E) non-specific adhesive events; (C and F) non-adhesive events. Curves are offset by the two axes.
